# Severe Pulmonary Hypertension in COPD: Impact on Survival and Diagnostic Approach

**DOI:** 10.1016/j.chest.2022.01.031

**Published:** 2022-01-31

**Authors:** Gabor Kovacs, Alexander Avian, Gerhard Bachmaier, Natascha Troester, Adrienn Tornyos, Philipp Douschan, Vasile Foris, Teresa Sassmann, Katarina Zeder, Jörg Lindenmann, Luka Brcic, Michael Fuchsjaeger, Alvar Agusti, Horst Olschewski

**Affiliations:** a Department of Pulmonology, University Clinic of Internal Medicine, Graz, Austria; b Ludwig Boltzmann Institute for Lung Vascular Research, Graz, Austria; c Institute for Medical Informatics, Statistics and Documentation, Graz, Austria; d Division of General Radiology, Department of Radiology, Graz, Austria; e Department of Thoracic and Hyperbaric Surgery, University Clinic of Surgery, Graz, Austria; f Institute for Pathology, Medical University of Graz, Graz, Austria; g Respiratory Institute, Hospital Clinic, Universitat de Barcelona, IDIBAPS, Ciberes, Spain

**Keywords:** COPD, heart catheterization, pulmonary hypertension

## Abstract

**Background:**

Severe pulmonary hypertension (PH) is prognostically highly relevant in patients with COPD. The criteria for severe PH have been defined based on hemodynamic thresholds in right heart catheterization.

**Research Question:**

Can noninvasive clinical tools predict severe PH in patients with COPD? How does the mortality risk change with increasing severity of airflow limitation and pulmonary vascular disease?

**Study Design and Methods:**

We retrospectively analyzed all consecutive patients with COPD with suspected PH undergoing in-depth clinical evaluation, including right heart catheterization, in our PH clinic between 2005 and 2018. Clinical variables potentially indicative of severe PH or death were analyzed using univariate and stepwise multivariate logistic regression and Cox regression analysis adjusted for age and sex.

**Results:**

We included 142 patients with median FEV_1_ of 55.0% predicted (interquartile range [IQR], 42.4%–69.4% predicted) and mean pulmonary arterial pressure of 35 mm Hg (IQR, 27–43 mm Hg). A multivariate model combining echocardiographic systolic pulmonary arterial pressure of ≥ 56 mm Hg, N-terminal pro-brain natriuretic peptide (NT-proBNP) plasma levels of ≥ 650 pg/mL, and pulmonary artery (PA) to ascending aorta (Ao) diameter ratio on chest CT scan of ≥ 0.93 predicted severe PH with high positive and negative predictive values (both 94%). After correction for age and sex, both airflow limitation (*P* = .002; Global Initiative for Chronic Obstructive Lung Disease [GOLD] stages 1-2 vs stage 3: hazard ratio [HR], 1.56 [95% CI, 0.90-2.71]; GOLD stages 1-2 vs stage 4: HR, 3.45 [95% CI, 1.75-6.79]) and PH severity (*P* = .012; HR, 1.85 [95% CI, 1.15-2.99]) remained associated independently with survival. The combination of GOLD stages 3 and 4 airflow limitation and severe PH showed the poorest survival (HR for death, 3.26 [95% CI, 1.62-6.57; *P* = .001] vs GOLD stages 1-2 combined with nonsevere PH).

**Interpretation:**

In patients with COPD, the combination of echocardiography, NT-proBNP level, and PA to Ao diameter ratio predicts severe PH with high sensitivity and specificity. The contribution of severe PH and severe airflow limitation to impaired survival is comparable.

## Graphical Abstract

**Figure:**
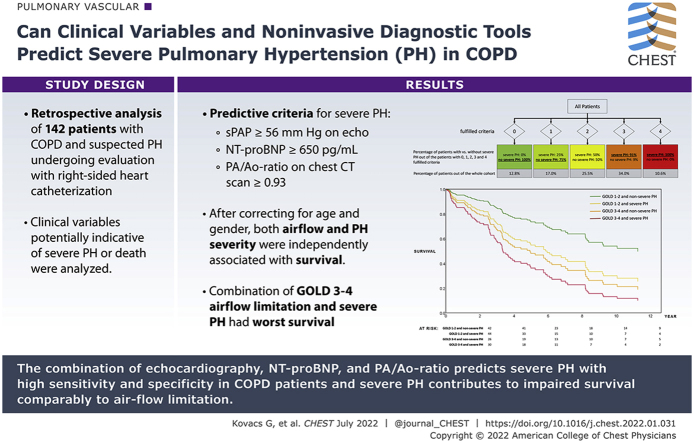

**Take-home Points**

**Study Question:** Can noninvasive clinical tools predict severe pulmonary hypertension (PH) in patients with COPD? How does the mortality risk change with increasing severity of airflow limitation and pulmonary vascular disease?

**Results:** The combination of echocardiographic systolic PAP of ≥ 56 mm Hg, N-terminal pro-brain natriuretic peptide (NT-proBNP) plasma levels of ≥ 650 pg/mL, and main pulmonary artery (PA) to ascending aorta (Ao) diameter ratio on chest CT scan of ≥ 0.93 predicted severe PH with high positive and negative predictive values. Both airflow limitation and PH severity were associated independently with survival.

**Interpretation:** In patients with COPD, the combination of echocardiography, NT-proBNP level, and PA to Ao diameter ratio predicts severe PH with high sensitivity and specificity. The contribution of severe PH and severe airflow limitation to impaired survival is comparable.

Pulmonary arterial pressure (PAP) often is increased moderately in patients with COPD.^1^ Severe pulmonary hypertension (PH) is present in 1% to 4% of patients^2^, ^3^, ^4^, ^5^, ^6^ and may represent a pulmonary vascular phenotype of COPD.^7^ Such a phenotype may justify PAH therapy on an individual basis.^1^^,^^8^ Given the high prevalence of COPD, probably many thousands of patients with COPD worldwide harbor severe PH, outnumbering patients with pulmonary arterial hypertension (PAH).^7^

The 6th World Symposium on Pulmonary Hypertension (WSPH) proposed hemodynamic thresholds for the classification of COPD with no PH, mild to moderate PH, and severe PH.^1^ However, little information is available regarding which clinical variables and noninvasive clinical tools predict the presence of severe PH in COPD, that is, which patients with COPD have a high risk of severe PH and should undergo diagnostic right heart catheterization (RHC). In addition, little is known about the prognostic relevance of the proposed stratification and how the severity of airflow limitation and pulmonary vascular disease contribute to mortality.

Accordingly, this study sought to investigate (1) which clinical variables and noninvasive diagnostic tools predict the presence of severe PH in COPD and (2) what the effect is of increasing airflow limitation and pulmonary vascular disease on mortality. Because all clinical thresholds that currently are used to define the severity of pulmonary vascular disease (including the suggested threshold for severe PH) and airflow limitation are based on expert opinion rather than evidence, we included patients representing the entire spectrum of pulmonary vascular disease (from normal hemodynamics to severe PH) and airflow limitation (from mild to severe obstructive airway disease).

## Study Design and Methods

### Study Design and Ethics

This was a retrospective analysis of all consecutive patients with COPD who underwent clinically indicated RHC for suspected PH at our clinic between March 2005 and January 2018. The diagnosis of COPD and the severity of airflow limitation were established according to the Global Initiative for Chronic Obstructive Lung Disease (GOLD) recommendations^9^ by two independent respiratory physicians. Regarding the PH classification, we used the following criteria: only patients with COPD with no other plausible cause for PH were placed in the group 3 PH category; in case of pulmonary arterial wedge pressure of > 15 mm Hg, the patient was placed in group 2 PH category; in case of significant abnormalities on the V̇/Q̇ scan, the CT imaging pulmonary angiogram, or both, the patient was placed in the chronic thromboembolic PH category. In the presence of disorders listed for group 5 PH (eg, sarcoidosis), the patient was placed in the group 5 PH category. In the resting patients, in case of mild airflow obstruction (FEV_1_ > 60% predicted) and no severe changes in the thin-slice CT scan investigation, the patient was placed in the PAH with concomitant COPD category. The study was approved by the institutional ethics board (Identifier: EK: 32-180 ex 19/20).

### Patients and Measurements

At the time of RHC, patients were carefully clinically evaluated. The complete dataset resulting from these investigations was included into a local database, the Graz Pulmonary Hypertension in COPD registry. A detailed list of parameters appears in e-Table 1.

Routine RHC was performed in all patients as described previously.^10^ For zero reference, the midthoracic level was used. Pressures were recorded continuously and averaged over several respiratory cycles during spontaneous breathing. Cardiac output was determined by thermodilution. The severity of PH was assessed according to the recommendations of the 6th WSPH.^1^ Specifically, (1) COPD with severe PH was defined as mean PAP (mPAP) of ≥ 35 mm Hg or mPAP of ≥ 25 mm Hg with low cardiac index (< 2.0 L/min/m^2^), (2) COPD with PH (referred to as moderate PH herein) was defined as mPAP between 25 and 34 mm Hg or mPAP between 21 and 24 mm Hg with pulmonary vascular resistance of ≥ 3 Wood units, and finally, (3) COPD without PH was defined as mPAP of < 21 mm Hg or mPAP of 21 to 24 mm Hg with pulmonary vascular resistance of < 3 Wood units.

### Statistical Analysis

Results of continuous variables are presented as mean ± SD or median (interquartile range [IQR]), whereas categorical data are shown as absolute and relative frequency. Clinical parameters potentially indicative of the presence of severe PH were analyzed first using univariate logistic regression analysis. Therefore, the group of patients with severe PH was compared with the group without severe PH (ie, either with no PH or with mild to moderate PH). Stepwise multivariate logistic regression analysis then was performed including all variables that showed a *P* value of ≤ .05 in the univariate analysis. For the predictors of severe PH in the final model, the best cutoff score according to the Youden index was calculated. Sensitivity, specificity, negative predictive value, and positive predictive value for severe PH comparing the groups with values less than and more than the defined thresholds were calculated.

Survival was calculated from the date of the initial RHC to death (all-cause mortality) or lung transplantation. Clinical parameters and baseline characteristics potentially affecting survival in the entire population were analyzed using Cox regression analysis. These analyses were adjusted for age and sex. Before the analysis, the assumption of proportional hazard ratios (HRs) was checked. A stepwise multivariate Cox regression analysis including those variables that showed a *P* value of ≤ .05 was performed. In addition, a similar analysis was carried out in the subgroup of patients with severe PH.

Finally, patients with and without severe PH and patients who were and were not receiving PAH medications were compared using the *t* test or Mann-Whitney *U* test in case of continuous variables and using the χ ^2^ test or Fisher exact test in case of categorical data. Age- and sex-adjusted survival (death or lung transplantation) of patients who were and were not receiving PAH medications was compared using Cox regression analysis. A *P* value of < .05 was considered significant.

## Results

### Patient Characteristics

We included 142 patients with COPD in the analysis. Their clinical and hemodynamic characteristics are presented in Tables 1 and 2, respectively. Slightly more males (56%) were included in the study, and 61% of patients showed mild to moderate airflow limitation. When stratified for severity of PH, 23 patients demonstrated no PH, 45 demonstrated moderate PH, and 74 demonstrated severe PH. Of these, 71 showed mPAP of ≥ 35 mm Hg and just three showed mPAP of 25 to 34 mm Hg with low cardiac index. According to our strict classification rules, of the 119 patients with PH, 58 patients (49%) demonstrated group 3 PH, whereas the other patients were classified as demonstrating other PH forms (Table 1).

**Table 1:** Patient Characteristics (n = 142)

Characteristic	Data
Age, y	68.1 (61.7-73.1)
Height, cm	170 (165-176)
Weight, kg	74 (62-87)
BMI, kg/m^2^	25.6 (21.5-29.8)
Sex	
Male	79 (56)
Female	63 (44)
BP, mm Hg	
Systolic	124 (113-143)
Diastolic	66 (58-76)
Heart rate	73 (65-84)
WHO functional class^a^	
I	0 (0.0)
II	47 (33.6)
III	86 (61.4)
IV	7 (5.0)
Pulmonary function test	
FEV_1_, % predicted	55.0 (42.4-69.4)
FVC, % predicted	74.9 (59.9-90.1)
FEV_1_ to FVC ratio, %	60.3 (52.7-66.1)
TLC, % predicted	103.6 (92.6-118.9)
Dlco, % predicted	
cSB	56.4 (39.9-68.4)
cVA	68.1 (48.5-84.0)
GOLD stage of obstruction	
I	18 (12.7)
II	68 (47.9)
III	40 (28.2)
IV	16 (11.3)
Smoking history^a^	…
Current smoker	17 (12)
Pack-years	13 (0-30)
Laboratory parameters	
NT-proBNP, pg/mL	827 (254-2636)
C-reactive protein, mg/L	4.4 (1.9-11.7)
Echocardiography	
Estimated SPAP, mm Hg	60 (52-79)
TAPSE, mm	19 (16-23)
Blood gas analysis	
Arterialized capillary Po_2_, mm Hg	62.4 (56.8-69.8)
Arterialized capillary Pco_2_, mm Hg	37.9 (34.2-42.4)
Arterialized capillary oxygen saturation, %	93.6 (91.1-95.2)
Oxygen therapy	53 (37)
Dose, L/min	2.0 (2.0-3.0)
Chest CT scan	
Mean PA to Ao diameter ratio	0.98 (0.82-1.08)
Mean PA diameter, mm	33.8 (29.7-37.2)
Pulmonary hemodynamics	
mPAP, mm Hg	35 (27-43)
PAWP, mm Hg	10 (8-13)
PVR, WU	4.3 (2.9-7.4)
Cardiac index, L/min/m^2^	2.57 (2.17-3.07)
Systemic vascular resistance index, dyn/s/cm^5^/m^2^	2396 (1951-2801)
Severity of pulmonary hypertension	
None	23 (16)
Moderate	45 (32)
Severe	74 (52)
Exercise capacity	
6MWD, m	306 (213-387)
Peak oxygen uptake, % predicted	50 (37-67)
Clinical classification of PH	
No PH	23 (16)
PAH	17 (12)
Group 2 PH	21 (15)
Group 3 PH^b^	58 (41)
Chronic thromboembolic PH	9 (6)
Group 5 PH and combinations	14 (10)

Data are presented as No. (%) or median (interquartile range). 6MWD = 6-min walk distance; Ao = ascending aorta; cSB = single breath corrected for hemoglobin; cVA = alveolar volume corrected for hemoglobin; Dlco = diffusion capacity of lung for carbon monoxide; GOLD = Global Initiative for Chronic Obstructive Lung Disease; mPAP = mean pulmonary arterial pressure; NT-proBNP = N-terminal pro-brain natriuretic peptide; PA = pulmonary artery; PAWP = pulmonary arterial wedge pressure; PH = pulmonary hypertension; PVR = pulmonary vascular resistance; SPAP = systolic pulmonary arterial pressure; TAPSE = tricuspid annular plane systolic excursion; TLC = total lung capacity; WHO = World Health Organization; WU = Wood unit.

a Available for 140 patients.

b Among the 58 patients who were in the group 3 PH classification, 8 showed some additional fibrotic changes in the lung parenchyma, 7 had received a diagnosis of OSA, and 4 had received diagnoses of alveolar hypoventilation disorders in addition to COPD.

**Table 2:** Characteristics for Patients With COPD Without PH, With Moderate PH, and With Severe PH (N = 142)

Characteristic	COPD Without PH (n = 23)	COPD With Moderate PH (n = 45)	COPD With Severe PH (n = 74)	*P* Value
Age, y	67.7 (61.4-76.8)	68.8 (59.9-73.1)	68.0 (62.7-72.7)	.864
Height, cm	169 (161-172)	168 (164-175)	170 (167-177)	.068
Weight, kg	58.0 (53.0-80.0)	70.0 (62.0-78.0)	80.5 (66.0-96.0)	< .001
BMI, kg/m^2^	22.3 (19.1-28.9)	24.5 (21.2-27.6)	28.2 (23.1-32.2)	.001
Sex				
Male	8 (34.8)	22 (48.9)	49 (66.2)	.016
Female	15 (65.2)	23 (51.1)	25 (33.8)	…
BP, mm Hg				
Systolic	124 (117-147)	125 (114-139)	120 (112-143)	.236
Diastolic	65 (58-72)	67 (60-76)	66 (56-76)	.561
Heart rate	71 (61-79)	77 (65-89)	71 (66-84)	.167
WHO functional class^a^				< .001
I	0 (0.0)	0 (0.0)	0 (0.0)	
II	16 (72.7)	14 (31.1)	17 (23.3)	
III	5 (22.7)	30 (66.7)	51 (69.9)	
IV	1 (4.5)	1 (2.2)	5 (6.8)	
Pulmonary function test				
FEV_1_, % predicted	59.0 (48.4-76.4)	52.9 (32.1-68.5)	55.0 (44.4-66.2)	.179
FVC, % predicted	82.9 (61.9-94.7)	80.2 (53.0-90.7)	73.5 (61.1-84.9)	.436
FEV_1_ to FVC ratio, %	61.0 (57.0-66.1)	58.2 (47.4-64.0)	61.8 (53.1-66.8)	.045
TLC, % predicted	109.2 (103.4-140.1)	111.4 (96.0-135.1)	96.7 (87.0-111.6)	< .001
Dlco, % predicted				
cSB	60.3 (46.4-68.0)	51.7 (39.5-64.9)	56.6 (37.0-72.0)	.266
cVA	78.4 (57.5-95.6)	60.6 (44.3-74.2)	71.2 (48.9-88.5)	.048
GOLD stage of obstruction				.119
I	5 (21.7)	6 (13.3)	7 (9.5)	
II	13 (56.5)	18 (40.0)	37 (50.0)	
III	2 (8.7)	13 (28.9)	25 (33.8)	
IV	3 (13.0)	8 (17.8)	5 (6.8)	
Smoking history^a^				
Current smoker	3 (13.0)	5 (11.4)	9 (12.3)	.978
Pack-years	9 (0-40)	15 (0-30)	11 (0-33)	.646
Laboratory parameters				
NT-proBNP, pg/mL	277 (111-567)	408 (176-1,126)	1,921 (752-3,391)	< .001
C-reactive protein, mg/L	1.9 (1.0-6.6)	4.2 (1.9-12.0)	5.5 (2.7-12.1)	.044
Echocardiography				
Estimated SPAP, mm Hg	45 (36-54)	55 (48-64)	70 (60-82)	< .001
TAPSE, mm	24 (20-25)	21 (16-23)	18 (15-21)	.060
Blood gas analysis				
Arterialized capillary Po_2_, mm Hg	71.4 (64.9-77.7)	61.0 (57.3-69.4)	60.4 (55.2-66.6)	< .001
Arterialized capillary Pco_2_, mm Hg	37.2 (34.4-40.5)	39.7 (36.5-44.7)	37.4 (33.4-42.3)	.383
Arterialized capillary oxygen saturation, %	95.5 (93.7-96.9)	93.5(91.6-95.4)	92.8 (89.9-94.5)	< .001
Oxygen therapy	4 (17)	17 (38)	32 (43)	.076
Dose, L/min	2.0 (1.75-2.0)	2.0 (2.0-3.0)	2.0 (2.0-4.0)	…
Chest CT scan				
Mean PA to Ao diameter ratio	0.76 (0.72-0.91)	0.89 (0.83-1.06)	1.03 (0.91-1.14)	< .001
Mean PA diameter, mm	27.5 (23.9-31.7)	32.4 (28.5-35.6)	36.0 (33.1-40.0)	< .001
Pulmonary hemodynamics				
mPAP, mm Hg	19 (15-23)	28 (27-32)	43 (38-50)	< .001
PAWP, mm Hg	8 (6-9)	9 (7-12)	11 (8-15)	< .001
PVR, WU	2.38 (1.92-2.86)	3.59 (2.85-4.37)	6.97 (4.69-9.02)	< .001
Cardiac index, L/min/m^2^	2.68 (2.34-3.08)	2.68 (2.36-3.14)	2.44 (1.98-2.87)	.008
Systemic vascular resistance index, dyn/s/cm^5^/m^2^	2,563 (1,921-3,189)	2,312 (20,290-2,673)	2,409 (1,948-3,075)	.737
Exercise capacity				
6MWD, m	350 (281-416)	337 (265-417)	272 (186-360)	.005
Peak oxygen uptake, % predicted	75 (63-85)	53 (43-73)	39 (31-52)	< .001
Survival, %				
1 y	100.0	91.1	86.4	…
3 y	90.7	75.2	64.2	…
5 y	78.6	55.9	48.1	…

Data are presented as No. (%) or median (interquartile range), unless otherwise indicated. Ao = ascending aorta; cSB = single breath corrected for hemoglobin; cVA = alveolar volume corrected for hemoglobin; Dlco = diffusion capacity of lung for carbon monoxide; GOLD = Global Initiative for Chronic Obstructive Lung Disease; mPAP = mean pulmonary arterial pressure; NT-proBNP = N-terminal pro-brain natriuretic peptide; PA = pulmonary artery; PAWP = pulmonary arterial wedge pressure; PH = pulmonary hypertension; PVR = pulmonary vascular resistance; 6MWD = 6-min walk distance; SPAP = systolic pulmonary arterial pressure; TAPSE = tricuspid annular plane systolic excursion; TLC = total lung capacity; WHO = World Health Organization; WU = Wood unit.

a Available for 140 patients.

### Prediction of Severe PH

**Figure 1:**
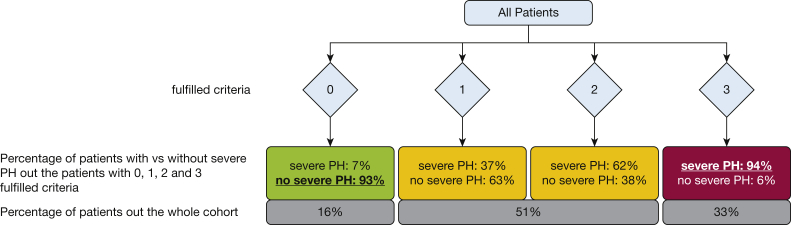
Diagram showing probability of severe PH in patients with COPD fulfilling 0 to 3 criteria: systolic pulmonary arterial pressure, estimated by echocardiography with a threshold of ≥ 56 mm Hg, N-terminal pro-brain natriuretic peptide with a threshold of ≥ 650 pg/mL, and the main pulmonary artery to the ascending aorta diameter ratio at the tubular site on chest CT imaging with a threshold of ≥ 0.93. In addition, the proportion of patients in the COPD population fulfilling 0, 1 to 2, and all 3 criteria is shown. PH = pulmonary hypertension.

In the univariate analysis, a large number of noninvasive clinical parameters were associated with severe PH (e-Table 2). Based on multivariate analysis, we included three independent predictive variables into our model: (1) systolic PAP (sPAP) of ≥ 56 mm Hg, estimated by echocardiography; (2) N-terminal pro-brain natriuretic peptide (NT-proBNP) plasma levels of ≥ 650 pg/mL); and (3) the main pulmonary artery (PA) to ascending aorta (Ao; at the tubular site) diameter ratio on chest CT imaging was ≥ 0.93. The positive predictive value increased proportional to the number of positive criteria in a given patient. When all criteria were met (33% of patients), the specificity and the positive predictive value were 94.9% and 93.5% for severe PH, respectively. The presence of at least one of the criteria (84% of patients) showed a sensitivity of 98.2%. By contrast, if none of the criteria were present (16% of patients), the probability of severe PH was only 6.7% (Fig 1). In terms of negative and positive predictive values, as well as sensitivity and specificity, this composite multivariate prediction model outperformed echocardiography (for the sPAP ≥ 56-mm Hg cutoff: negative predictive value, 76%; positive predictive value, 77%; sensitivity, 86%; specificity, 64%) for the diagnosis of severe PH.

Interestingly, among patients with severe PH, BMI was elevated and a male preponderance was found (Table 2). Although because of multicolinearities we did not include BMI into the original model, we developed a second model to predict the presence of severe PH in COPD by adding BMI with a threshold of ≥ 28.4 kg/m^2^ to the three variables described previously. According to this model, every patient with COPD in the cohort in whom all four criteria were met (n = 12) was placed in the severe PH category, and no patient with COPD showed severe PH in whom none of the criteria were met (n = 10) (e-Fig 1). The low number of patients in these groups precludes reliable statistical analysis, but this approach may deserve further exploration in larger patient cohorts.

### Survival

Median follow-up time was 3.7 years (IQR, 2.2-7.1 years; end of follow-up, December 31, 2018). Overall, 72 patients (50.7%) died during follow-up and three underwent lung transplantation (2.1%). No patient was lost to follow-up. The median time to the combined end point of death or lung transplantation was 5.9 years (95% CI, 4.2-7.7 years) from baseline.

**Figure 2:**
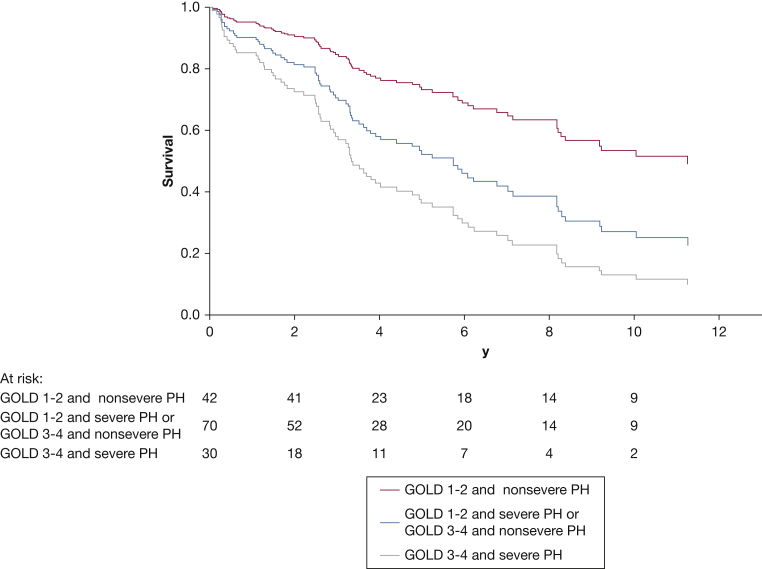
Survival curves for patients with COPD based on severity of airflow limitation and PH. Red curve indicates good prognosis, consisting of GOLD stages 1 or 2 and no or moderate PH. Blue curve indicates intermediate prognosis, including either GOLD stages 3 or 4 or severe PH. Gray curve indicates poor prognosis, with the combination of GOLD stages 3 or 4 and severe PH. Curves are based on a Cox proportional hazard model. GOLD = Global Initiative for Chronic Obstructive Lung Disease; PH = pulmonary hypertension.

In the entire population, after adjusting for age and sex in the multivariate model, both airflow limitation (*P* = .002; GOLD stages 1-2 vs stage 3: HR, 1.56 [95% CI, 0.90-2.71]; GOLD stages 1-2 vs stage 4: HR, 3.45 [95% CI, 1.75-6.79]) and PH severity (*P* = .012; HR, 1.85 [95% CI, 1.15-2.99]) were associated independently with survival. When the combination of airflow limitation (GOLD stages 1-2 vs stages 3-4) and PH (nonsevere vs severe) was analyzed, three distinct groups with different prognoses (*P* = .004) could be identified (Fig 2, e-Table 3): (1) good prognosis, consisting of GOLD stages 1 or 2 and nonsevere PH (3-year survival, 90% [95% CI, 80-99]); (2) intermediate prognosis, including either GOLD stages 3 or 4 or severe PH (HR vs first group, 2.09 [95% CI, 1.14-3.83; *P* = .017]; 3-year survival, 68% [95% CI, 57%-79%]); and (3) poor prognosis, with the combination of GOLD stages 3 or 4 and severe PH (HR vs first group, 3.26 [95% CI, 1.62-6.57; *P* = .001]; 3-year survival, 54% [95% CI, 36-73]). e-Figure 2 depicts survival when the COPD population is divided into four groups by showing patients with GOLD stages 1 or 2 and severe PH as well as patients with GOLD stages 3 or 4 and no or moderate PH separately. e-Table 4 provides the results of univariate and multivariate analysis for GOLD stage and PH severity as significant predictors of survival.

e-Table 5 presents the hemodynamic, pulmonary function, and exercise parameters related to survival in the univariate analysis in patients with COPD with severe PH. Many of these factors were associated significantly with survival, but most of them were correlated strongly with each other. In the multivariate analysis, only 6-min walk distance (*P* < .001) and systemic vascular resistance index (*P* = .018) remained significant independent predictors of prognosis. For these parameters, the best prognostic cutoffs for a poor prognosis were 6-min walk distance of < 200 m and systemic vascular resistance index of < 2,800 dyn/s/cm^5^/m^2^.

### Impact of PAH-Targeted Therapy

Before the baseline RHC at our clinic, 9% of patients already were receiving a PAH medication (ie, introduced by another center). After the baseline RHC, 44% of patients (61% of patients with severe PH vs 27% of patients with moderate PH; *P* < .001) received a PAH drug or oral combination therapy. Patients receiving PAH treatment showed less severe airflow limitation, more severe hemodynamic impairment, and more severe limitation of exercise capacity as compared with the other patients (e-Table 6). Although baseline hemodynamics were worse in the treated vs untreated patients, survival was not significantly different between these groups in the entire cohort (HR, 1.13 [95% CI, 0.72-1.78]; *P* = .601) (e-Fig 3) and in patients with severe PH (HR, 0.76 [95% CI, 0.41-1.40]; *P* = .376) (e-Fig 4).

## Discussion

In this study, we aimed to search for clinical variables and noninvasive diagnostic tools that may predict the presence of severe PH in COPD and to investigate the effect of increasing airflow limitation and pulmonary vascular disease on mortality. We included all patients undergoing RHC who met the criteria for COPD, independent of the putative pathologic mechanism for PH. This approach was used because in many patients with COPD, it is difficult to decide on the dominant pathologic mechanism for PH. Particularly the decision between group 1 and group 3 PH proved to be demanding. We found that among patients with COPD, (1) criteria for severe PH mostly were based on mPAP of ≥ 35 mm Hg and rarely on mPAP of 25 to 34 mm Hg in combination with cardiac index of < 2.0 L/min/m^2^; (2) a simple, noninvasive clinical assessment using sPAP by echocardiography, NT-proBNP level, and the PA to Ao diameter ratio by chest CT scan predicts severe PH with high sensitivity and specificity; and (3) prognosis is limited independently by both the severity of airflow limitation and the severity of PH and that these factors are approximately equipotent.

### Previous Studies

One of the largest cohorts analyzed for the clinical relevance of pulmonary vascular disease in COPD was the Assessing the Spectrum of Pulmonary Hypertension Identified at a Referral Centre (ASPIRE) registry, published in 2013.^11^ In that study, 101 patients with group 3 PH and a mean follow-up time of 2.3 years were included. Patients with severe PH (defined as mPAP of ≥ 40 mm Hg) showed significantly worse survival as compared with patients with mild to moderate PH (mPAP, 25-39 mm Hg). This study provides important additional information. To our knowledge, since the introduction of the current clinical classification of PH, this is the first COPD cohort to have undergone RHC and long-term follow-up in which patients with COPD representing the entire spectrum of pulmonary vascular involvement (from normal hemodynamics to severe PH) and airflow limitation (from mild to severe obstructive airway disease) meeting criteria for WSPH categories other than group 3 PH were included. Therefore, this population is likely to be more representative of a real-world setting and may allow the combined assessment of the prognostic relevance of airflow limitation and PH. In addition, this cohort is the first in which stratification was used as suggested in the latest WSPH proceedings.^1^ Therefore, this study may help to develop practical management strategies for patients with COPD with a large spectrum of pulmonary vascular disease and airflow obstruction.

### Interpretation of Findings

#### Patient Heterogeneity

A relevant finding of our study is the heterogeneity of patients with COPD regarding the putative pathologic mechanism underlying the mPAP increase. According to the 6th WSPH proceedings, pulmonary function testing and CT scan morphologic features may help to categorize patients as group 1 PH (PAH) or group 3 PH. The presence of FEV_1_ of < 60% predicted or extensive CT scan parenchymal damage supports the diagnosis of group 3 PH, whereas their absence suggests group 1 PH. However, in patients with COPD, for example, heart disease (mainly heart failure with preserved ejection fraction), collagen vascular disease, or chronic thromboembolic disease also may be present and may result in elevated mPAP. Applying our classification rules (see Methods), we ended up with only about half of the included 119 patients with PH in the group 3 PH category and the other half mainly in the PAH, chronic thromboembolic PH, or group 2 PH categories (14%, 8%, and 18%, respectively) (Table 1). This finding is in agreement with the fact that COPD is a common disease and frequently is associated with other comorbidities.^6^ Of note, in 18 of 21 patients with group 2 PH, heart failure with preserved ejection fraction was present.

Another relevant finding of this study is that the vast majority of patients meeting the criteria for severe PH showed mPAP of ≥ 35 mm Hg, and those fulfilling the cardiac index-based criterion only (mPAP of ≥ 25 mm Hg with cardiac index of < 2.0 L/min/m^2^) were rare (3/74 patients). This suggests that most patients with COPD and severe PH may be detected by the recognition of a severe PAP elevation.

#### Clinical Recognition of Severe PH in Patients With COPD

Although some studies have described echocardiographic and other variables predicting the presence of severe PH in patients with COPD,^12^ currently, no robust clinical algorithm is available for this purpose. We found that NT-proBNP level of ≥ 650 pg/mL, echocardiographic sPAP of ≥ 56 mm Hg, and PA to Ao diameter ratio of ≥ 0.93 on chest CT scan are instrumental to diagnose severe PH in patients with COPD. When a patient meets these three criteria, the probability of severe PH is 94% (29/31 patients) (Fig 1). This suggests that patients demonstrating all three criteria should be referred to an expert center for RHC and evaluation for individualized therapy. Even in patients demonstrating only two of these criteria, the probability of severe PH was 62%. However, the probability of severe PH in patients not fulfilling any of these criteria was low (7%; 1/15 patients), suggesting that referral and RHC may be indicated only if group 1 or group 4 PH are suspected. In an alternative model additionally including BMI, we could increase the sensitivity and specificity for severe PH in patients with zero or all four points; however, the number of patients with a clear prediction decreased (e-Fig 1).

Echocardiography is recommended as a preferred noninvasive tool to estimate pulmonary pressures in COPD,^1^ although significant limitations are present, particularly in patients with emphysema.^13^ Our data indicate that echocardiography is an important noninvasive diagnostic tool for detecting severe PH in COPD, but the diagnostic accuracy could be improved markedly when it was complemented by NT-proBNP level and the PA to Ao diameter ratio. NT-proBNP level also has been recognized as useful tool for the diagnosis of PH in patients with end-stage lung diseases referred for lung transplantation,^14^ but it may be strongly biased by left heart and renal failure and has not been validated for the identification of severe PH in COPD. PA to Ao diameter ratio also has been described in previous studies as a marker of PH,^15^^,^^16^ and the best cutoff, as in our study, was around 1.0. In one study, the method even outperformed echocardiography.^17^ In addition, this ratio was identified as a predictor of COPD exacerbations.^18^ However, it has not been used for detection of severe PH in COPD. Taken together, the three clinical parameters identified in our analysis predicting severe PH in COPD are supported by previous studies and seem to be robust and suitable for daily clinical use.

#### Prognostic Relevance of Severe PH in Patients With COPD

We found that the severity of both airflow limitation and pulmonary vascular disease contributed to increased mortality significantly, independently, and about equally. This finding supports the hypothesis that smoking, aging, and other factors triggering both endothelial and epithelial dysfunction cause a number of pathologic mechanisms that eventually limit life expectancy.^7^

In patients with COPD and severe PH, quite a number of variables were associated significantly with survival in the univariate analysis (e-Table 5). Multivariate analysis, however, identified only two independent predictors: 6-min walk distance, with a best predictive threshold at 200 m, and systemic pulmonary vascular resistance index, with the best predictive threshold at 2,800 dyn/s/cm^5^/m^2^. Of note, in this analysis, only patients with severe PH were included, which may explain why some previously described prognostic parameters for COPD or for group 3 PH, such as lung diffusion capacity for carbon monoxide^19^^,^^20^ or pulmonary artery enlargement,^18^^,^^21^, ^22^, ^23^ did not reach statistical significance. The fact that the absence of a high systemic pulmonary vascular resistance index was an independent predictor of poor prognosis in the multivariate analysis suggests that systemic vasodilation (eg, resulting from hypoxemia) may represent an important complication in these patients, which deserves further exploration. In addition, patients with COPD and severe PH demonstrated higher pulmonary arterial wedge pressure as compared with patients with no PH or nonsevere PH, suggesting that left heart disease (mainly heart failure with preserved ejection fraction) may be a relevant comorbidity in these patients.

#### Impact of PAH Therapy in Patients With COPD

Previous data revealed a specific histologic pattern of the pulmonary vasculature in patients with COPD and severe PH characterized by microvessel remodeling and decreased capillary density,^24^ suggesting important similarities with idiopathic PAH.^25^ Targeted PAH therapy is not recommended for group 3 PH,^8^ and such drugs may be indicated only in severe PH on an individual basis or in scientific studies. Because of an increased risk of adverse events, such treatments should be used only in experienced expert centers.^8^ Most of the beneficial effects of PAH drugs have been described in uncontrolled studies that mainly enrolled patients with severe PH.^26^, ^27^, ^28^, ^29^, ^30^, ^31^, ^32^

In the present cohort, PAH-targeted therapy was initiated more frequently in patients with severe PH as compared with patients with moderate PH (61% vs 27%). Although more severely impaired in terms of pulmonary hemodynamics and exercise capacity, patients receiving PAH-targeted treatment showed a similar survival as those receiving no such therapy, indicating a possible beneficial effect. This may support the hypothesis that a subgroup of patients with COPD and severe PH profits from PAH-targeted treatment. The potential beneficial effects of PAH treatment in patients with COPD and severe PH must be confirmed in prospective randomized controlled studies.

### Strengths and Limitations

To our knowledge, this study is among the COPD studies with the largest number of mortality events, in which all patients underwent comprehensive clinical evaluation and RHC. All clinical evaluations and technical measurements were carried out by a relatively small and constant team under stable external conditions. As a further strength of the study, the included patients represented the entire spectrum of pulmonary vascular involvement and airflow limitation. Therefore, our findings may help to develop practical management strategies for patients with COPD with a large spectrum of pulmonary vascular disease, and our data support that, regardless of cause, hemodynamic severity predicts survival in COPD. However, this strategy led to an increased heterogeneity of the groups, and because of this, some expected changes in patients with severe PH (eg, strongly decreased diffusion capacity for carbon monoxide) may not be visible.

We acknowledge several limitations of our study. First, this was a retrospective and monocentric study, and although the observation time was relatively long, the sample size and statistical power remain limited. Therefore, validation of our findings in independent cohorts will be needed. Second, our cohort of patients with COPD is enriched by patients with severe PH, making it more representative of a population seen in a PH clinic, rather than in a COPD clinic, in which severe PH may be present only in 1% to 4% of patients.^2^, ^3^, ^4^^,^^33^ This selection bias may limit the generalizability of our findings; however, this allowed for thorough analysis of the pulmonary vascular effects, which was the main focus of the study. Further, based on the characteristics of the patients, individuals with severe emphysema and air trapping may have been underrepresented in the cohort, and the use of a fixed FEV_1_ to FVC ratio instead of lower limits of normal spirometry to define obstruction might have overestimated obstructive disease in some elderly patients. Finally, our primary approach was not to search generally for variables predicting prognosis in COPD, but rather to investigate how mortality risk changes in patients with COPD with increasing severity of airflow limitation and pulmonary vascular disease. Therefore, established composite prognostic scores in COPD, such as the BODE index, were not included in our survival analysis.

## Interpretation

The severity of both airflow limitation and pulmonary vascular disease similarly and independently may contribute to impaired survival in patients with COPD. Severe PH is predicted by the combination of echocardiography, NT-proBNP levels, and chest CT scan-derived PA to Ao diameter ratio. Further studies to validate these findings are warranted.

## Supplementary Data

e-Online Data
