# Supplementary material for: Severe Pulmonary Hypertension in COPD: Impact on Survival and Diagnostic Approach
Source: Chest. 2022 Jan 31;162(1):202–12. doi: 10.1016/j.chest.2022.01.031 (PMC10808070; doi:10.1016/j.chest.2022.01.031)
Supplement: e-Online Data [file mmc1.pdf]

CHEST Online Supplement

# Severe Pulmonary Hypertension in COPD

## Impact on Survival and Diagnostic Approach

*Gabor Kovacs, MD; Alexander Avian, PhD; Gerhard Bachmaier, PhD; Natascha Troester, MD; Adrienn Tornyos, MD, PhD; Philipp Douschan, MD; Vasile Foris, MD, PhD; Teresa Sassmann, MD; Katarina Zeder, MD; Jörg Lindenmann, MD; Luka Brcic, PhD; Michael Fuchsjaeger, MD, PhD; Alvar Agusti, MD, PhD; and Horst Olschewski, MD*

CHEST 2022; 162(1):202-212

***Online supplements are not copyedited prior to posting and the author(s) take full responsibility for the accuracy of all data.***

**© 2022 AMERICAN COLLEGE OF CHEST PHYSICIANS.** Reproduction of this article is prohibited without written permission from the American College of Chest Physicians. See online for more details. **DOI:** 10.1016/j.chest.2022.01.031

**e-Figure 1.** Probability of severe PH in COPD patients fulfilling 0-4 criteria (sPAP, estimated by echocardiography with a threshold at ≥ 56 mmHg, N-terminal pro brain natriuretic peptide with a threshold at ≥ 650 pg/ml, the diameter ratio of the main pulmonary artery / ascending aorta at the tubular site in chest CT with a threshold at ≥ 0.93 and body mass index ≥ 28.4 kg/$m^2$). In addition, the proportion of patients in our COPD population fulfilling 0, 1, 2, 3 and all 4 criteria is shown.

(PH: pulmonary hypertension, sPAP: systolic pulmonary arterial pressure)

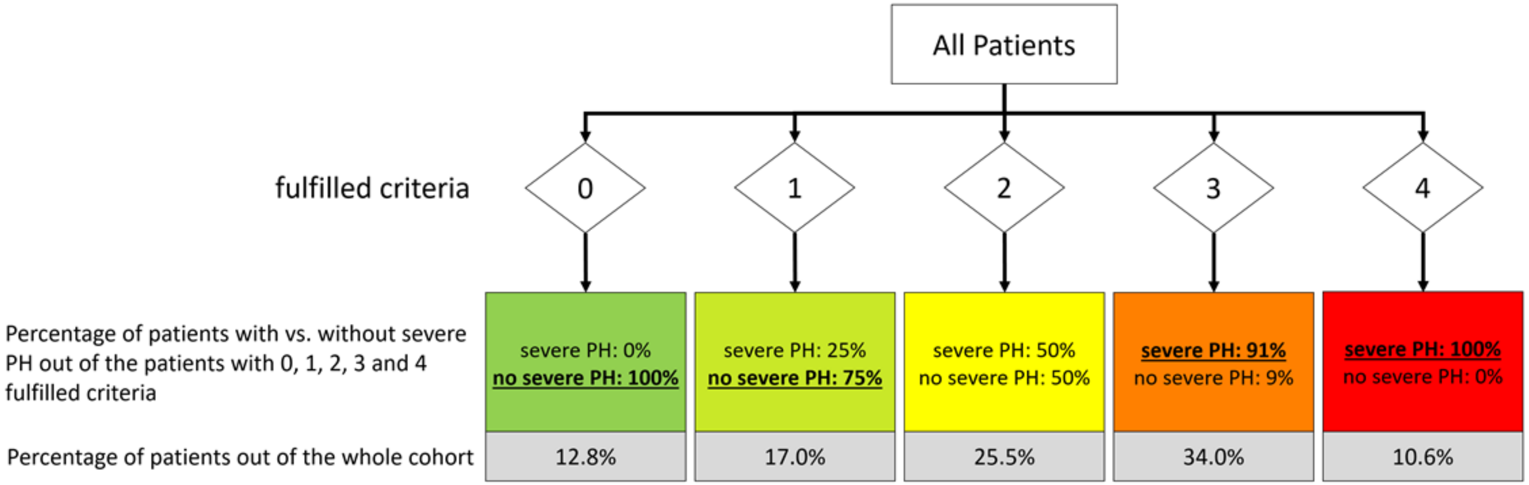


*Online supplements are not copyedited prior to posting and the author(s) take full responsibility for the accuracy of all data.*

**e-Figure 2.** Survival of COPD patients based on severity of airflow limitation and pulmonary hypertension (green curve: GOLD 1-2 and no or moderate PH; yellow curve: GOLD 1-2 and severe PH; orange curve: GOLD 3-4 and no or moderate PH; red curve: GOLD 3-4 and severe PH; curves are based on a cox proportional hazard model)

(PH: pulmonary hypertension)

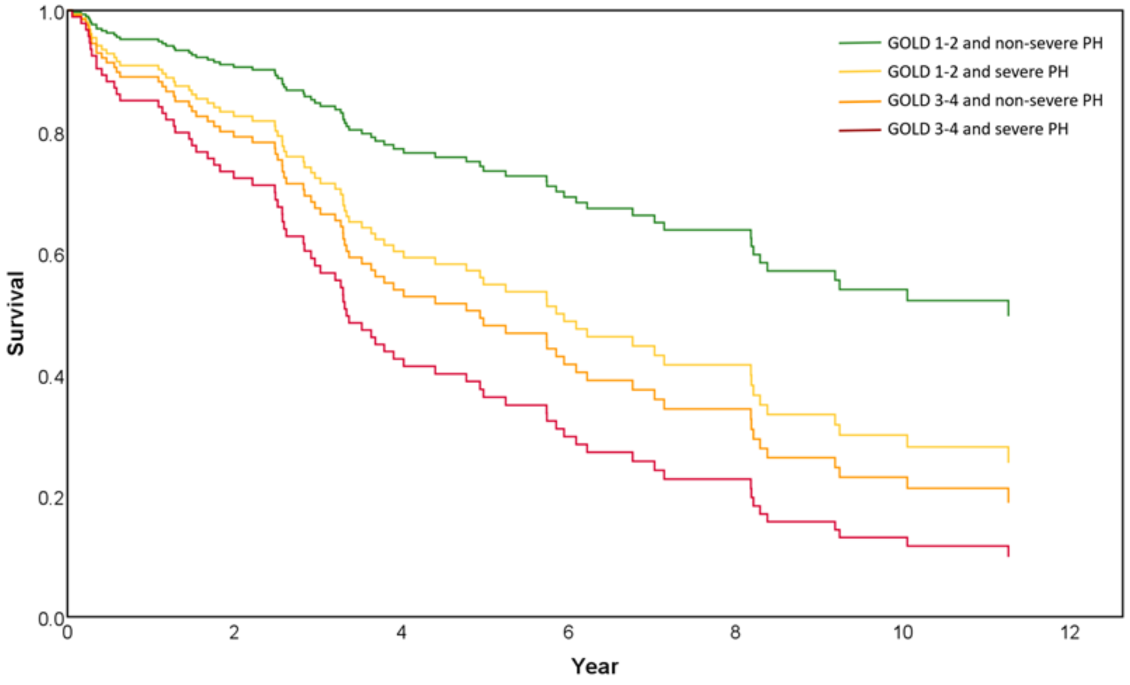


| At risk: | | | | | | |
|---|---|---|---|---|---|---|
| GOLD 1-2 and non-severe PH | 42 | 41 | 23 | 18 | 14 | 9 |
| GOLD 1-2 and severe PH | 44 | 33 | 15 | 10 | 7 | 4 |
| GOLD 3-4 and non-severe PH | 26 | 19 | 13 | 10 | 7 | 5 |
| GOLD 3-4 and severe PH | 30 | 18 | 11 | 7 | 4 | 2 |

*Online supplements are not copyedited prior to posting and the author(s) take full responsibility for the accuracy of all data.*

**e-Figure 3.** Survival of COPD patients with PH receiving (green curve) vs. not receiving (red curve) PAH treatment (curves are based on a cox proportional hazard model).

(PH: pulmonary hypertension, PAH: pulmonary arterial hypertension)

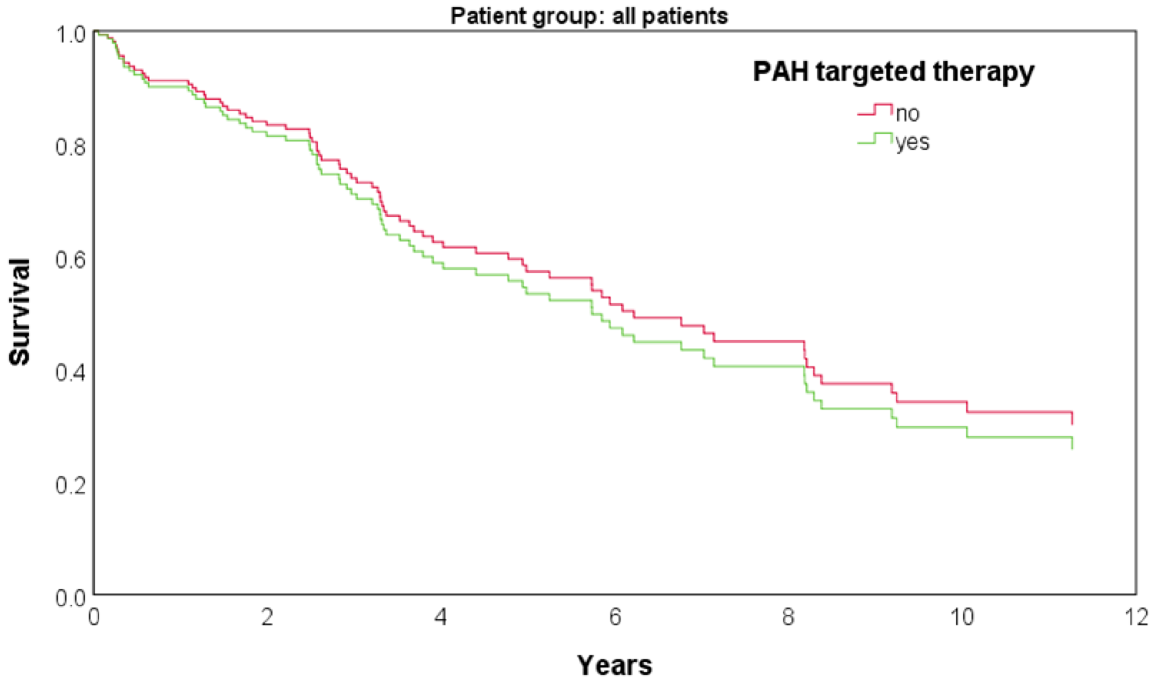

**e-Figure 4.** Survival of COPD patients with severe PH receiving (green curve) vs. not receiving (red curve) PAH treatment (curves are based on a cox proportional hazard model).

(PH: pulmonary hypertension, PAH: pulmonary arterial hypertension)

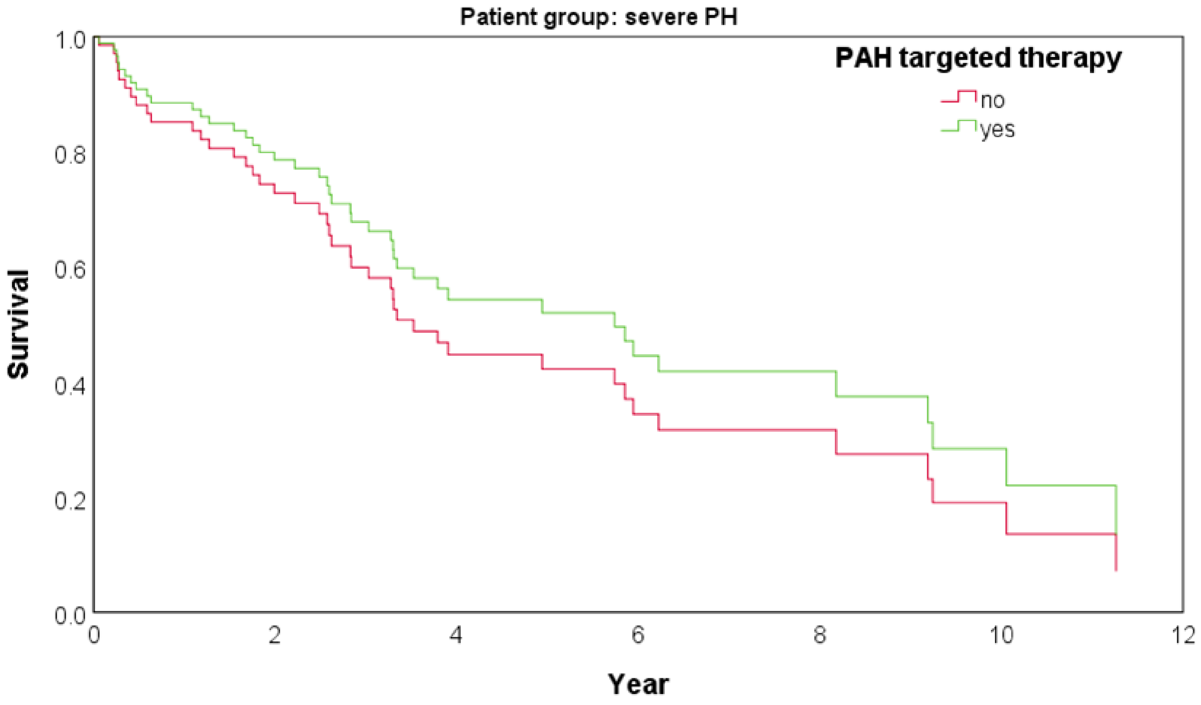

CHEST® Online Supplement

**e-Table 1. Patient characteristics used for analysis of diagnosis and prognosis of severe pulmonary hypertension**

**Patient characteristics (n=142)**: date of birth, date of death, reason of death, sex, height, weight, body mass index, body surface area

**Smoking history, Pack-years (n=140)**

**WHO functional class (n=140)**

**ECG variables (n=139)**: height of the R wave in lead I, height of the S wave in lead I, ratio of the R and S waves in lead I

**Laboratory parameters (n=139)**: hemoglobin, creatinine, uric acid, total bilirubin, N-terminal pro brain natriuretic peptide, C-reactive protein, INR, albumin, red cell distribution width, glomerular filtration rate

**Pulmonary Function Test variables (n=142)**: forced vital capacity, forced expiratory volume in the first second, ratio of forced expiratory volume in the first second and forced vital capacity, total lung capacity, single breath diffusion capacity of carbon monoxide corrected for hemoglobin, diffusion capacity of carbon monoxide corrected for hemoglobin and alveolar volume

**Blood gas analysis (n=142)**: arterialized capillary blood partial pressure of oxygen and partial pressure of carbon dioxide and oxygen saturation, $FiO_2$

**Echocardiographic variables (n=123)**: left ventricular ejection fraction, left ventricular end-diastolic diameter, end-diastolic diameter of the interventricular septum, end-diastolic diameter of the left ventricular posterior wall, fractional shortening, size of the left atrium, diameter of the aortic root, pulmonary arterial acceleration time, pulmonary arterial ejection time, left ventricular eccentricity index, size of the right atrium, size of the left ventricle, size of the right ventricle, transmitral E wave, transmitral A wave, transmitral deceleration time, E/E′, grade of tricuspid insufficiency, estimated right atrial pressure, estimated systolic pulmonary arterial pressure based on tricuspid regurgitation jet velocity and estimated right atrial pressure, tricuspid regurgitation jet velocity, grade of tricuspid insufficiency, grade of mitral insufficiency, grade of aortic insufficiency, grade of pulmonary insufficiency, grade of valvular stenoses, tricuspid annular plane systolic excursion, presence and diameter of pericardial effusion

**Right Heart Catheterization (n=142)**: heart rate, mean systemic arterial pressure, mean pulmonary arterial pressure, pulmonary arterial wedge pressure, right atrial pressure, cardiac output, systemic vascular resistance, pulmonary vascular resistance, cardiac index, systemic vascular resistance index, pulmonary vascular resistance index, ratio of pulmonary vascular resistance and systemic vascular resistance, systolic systemic blood pressure, diastolic systemic blood pressure, systolic pulmonary arterial pressure, diastolic pulmonary arterial pressure, mixed venous oxygen saturation, arterio-venous oxygen difference, oxygen uptake

**6 Minute Walk Distance (n=120)**

**Cardio Pulmonary Exercise Testing variables (n=51)**: peak oxygen uptake, heart rate reserve, breathing reserve, end-tidal carbon dioxide at rest and at peak exercise and the change between rest and exercise, alveolo-arterial difference of carbon dioxide at rest and at peak exercise, alveolo-arterial oxygen difference at rest and at peak exercise, minimal value of oxygen equivalent (EQO2), minimal value of carbon dioxide equivalent (EQCO2), nadir $Ve/VCO_2$, change in the partial pressure of carbon dioxide from rest to exercise, change in the partial pressure of oxygen from rest to exercise, change in base excess from rest to exercise

**Chest CT variables (n=109)**: main pulmonary artery diameter at the bifurcation, main pulmonary artery diameter at tubular site, ascending aorta diameter, ratio of the main pulmonary artery at tubular site and ascending aorta, antero-posterior diameter of the left atrium at the level of the aortic root, maximal antero-posterior diameter of the left atrium, maximal transversal diameter of the left atrium, right atrial diameter, ratio of transversal diameter of the right and left atrium

*Online supplements are not copyedited prior to posting and the author(s) take full responsibility for the accuracy of all data.*

**e-Table 2. Significant non-invasive variables to predict severe pulmonary hypertension (severe pulmonary hypertension vs. no severe pulmonary hypertension)**

| | univariable | | multivariable | |
|---|---|---|---|---|
| | OR (95%CI) | p value | OR (95%CI) | p value |
| **Patient characteristics**: | | | | |
| sex | 0.40 (0.20 - 0.79) | 0.009 | | |
| weight | 1.04 (1.02 – 1.07) | <0.001 | | |
| body mass index | 1.12 (1.05-1.19) | 0.001 | | |
| body surface area | 32.5 (5.3 – 198.5) | <0.001 | | |
| | | | | |
| **WHO functional class** | | | | |
| 2 | 1 | 0.008 | | |
| 3,4 | 2.67 (1.29 – 5.52) | | | |
| | | | | |
| **ECG variables**: | | | | |
| height of the R wave in lead I | 0.79 (0.66 - 0.93) | 0.005 | | |
| height of the S wave in lead I | 1.94 (1.51-2.49) | <0.001 | | |
| ratio of the R and S waves in lead I | 5.17 (2.63 – 10.17) | <0.001 | | |
| | | | | |
| **Laboratory parameters**: | | | | |
| uric acid | 1.22 (1.02 – 1.46) | 0.027 | | |
| total bilirubin | 4.74 (1.81 – 12.41) | 0.002 | | |
| N-terminal pro brain natriuretic peptide (natural logarithm of the NT pro BNP was used for the analysis) | 2.30 (1.68 – 3.13) | <0.001 | 1.79 (1.17 – 2.72) | 0.007 |
| red cell distribution width | 1.36 (1.12 – 1.67) | 0.002 | | |
| glomerular filtration rate | 0.986 (0.972 - 0.999) | 0.037 | | |
| | | | | |
| **Pulmonary Function Test variables**: | | | | |
| total lung capacity | 0.96 (0.94 - 0.98) | <0.001 | | |
| | | | | |
| **Blood gas analysis**: | | | | |
| Arterialized capillary partial pressure of oxygen | 0.94 (0.90 - 0.98) | 0.001 | | |
| Arterialized capillary oxygen saturation | 0.83 (0.74 - 0.93) | 0.002 | | |
| | | | | |
| **Echocardiography variables**: | | | | |
| fractional shortening | 0.94 (0.88 – 1.00) | 0.043 | | |
| size of the left atrium | 1.11 (1.02 – 1.20) | 0.011 | | |
| pulmonary arterial acceleration time | 0.97 (0.94 - 0.99) | 0.014 | | |
| grade of tricuspid insufficiency | | 0.202 | | |
| 0 or 1 | 1 | | | |
| 2 or 3 | 2.5 (0.61 – 10.23) | | | |

*Online supplements are not copyedited prior to posting and the author(s) take full responsibility for the accuracy of all data.*

| | | | | |
|---|---|---|---|---|
| estimated right atrial pressure | 1.29 (1.06 – 1.57) | 0.012 | | |
| estimated systolic pulmonary arterial pressure | 1.08 (1.05 – 1.11) | <0.001 | 1.06 (1.02-1.10) | 0.003 |
| | | | | |
| **6 Minute Walk Distance** | 0.995 (0.991 - 0.998) | 0.002 | | |
| | | | | |
| **Cardio Pulmonary Exercise Testing variables**: | | | | |
| peak oxygen uptake | 0.94 (0.91 - 0.98) | 0.001 | | |
| heart rate reserve | 0.97 (0.95 - 0.100) | 0.032 | | |
| end-tidal carbon dioxide at peak exercise | 0.47 (0.26 - 0.85) | 0.013 | | |
| alveolo-arterial difference of oxygen at rest | 1.07 (1.02 – 1.13) | 0.011 | | |
| alveolo-arterial difference of oxygen at peak exercise | 1.10 (1.03 – 1.16) | 0.002 | | |
| minimal value of oxygen equivalent (EQO2) | 1.17 (1.05 – 1.30) | 0.004 | | |
| minimal value of carbon dioxide equivalent (EQCO2) | 1.10 (1.02 -1.19) | 0.013 | | |
| change in the partial pressure of carbon dioxide from rest to exercise | 0.73 (0.58 - 0.93) | 0.009 | | |
| | | | | |
| **Chest CT variables**: | | | | |
| main pulmonary artery diameter at the bifurcation | 1.11 (1.04 – 1.18) | 0.001 | | |
| main pulmonary artery diameter at tubular site | | <0.001 | | |
| ratio of the main pulmonary artery at tubular site and ascending aorta | 133.1 (10.0 – 1722.5) | <0.001 | 45.8 (1.67 – 1256.3) | 0.024 |
| antero-posterior diameter of the left atrium at the level of the aortic root | | 0.024 | | |
| maximal antero-posterior diameter of the left atrium | 1.07 (1.02 – 1.12) | 0.006 | | |
| right atrial diameter | 1.10 (1.05 – 1.15) | <0.001 | | |
| ratio of transversal diameter of the right and left atrium | 14.86 (2.34 – 94.34) | 0.004 | | |

*Online supplements are not copyedited prior to posting and the author(s) take full responsibility for the accuracy of all data.*

**e-Table 3. 3 and 5 year survival rate (95% CI) and hazard ratio (HR) (95%CI) of COPD patients based on severity of airflow limitation and pulmonary hypertension**
(PH: pulmonary hypertension)

| | HR (95%CI) | 3 year | 5 year |
|---|---|---|---|
| Gold 1 or 2 / no or moderate PH | ref. | 0,90 (0,80-0,99) | 0,71 (0,55-0,86) |
| Gold 1 or 2 / severe PH or Gold 3 or 4 / no or moderate PH | 2.09 (1.14 – 3.83) | 0,68 (0,57-0,79) | 0,50 (0,37-0,62) |
| Gold 3 or 4 / severe PH | 3.26 (1.62 6.57) | 0,54 (0,36-0,73) | 0,45 (0,26-0,65) |

**e-Table 4. GOLD stage and PH severity as significant predictors of survival in COPD patients (n=142)**

| | univariable | | multivariable | |
|---|---|---|---|---|
| | HR (95%CI) | p value | HR (95%CI) | p value |
| | | | | |
| **PH** | | | | |
| non-severe (Ref) | 1 | 0.036 | 1 | 0.012 |
| severe | 1.65 (1.03 – 2.64) | | 1.85 (1.15 – 2.99) | |
| **GOLD Stage** | | 0.006 | | 0.002 |
| 1-2 (Ref) | 1 | | 1 | |
| 3 | 1.64 (0.94 – 2.85) | 0.080 | 1.56 (.90 – 2.71) | 0.110 |
| 4 | 2.92 (1.50 – 5.71) | 0.002 | 3.45 (1.75 – 6.79) | <0.001 |

*Online supplements are not copyedited prior to posting and the author(s) take full responsibility for the accuracy of all data.*

**e-Table 5. Significant predictors of survival in COPD patients with severe pulmonary hypertension in univariable analysis (n=74)**

| **Patient characteristics**: | HR (95%CI) | **p value** |
|---|---|---|
| age | | 0.022 |
| | | |
| **Basic clinical assessment** | | |
| heart rate | 1.04 (1.02 – 1.06) | 0.001 |
| | | |
| **WHO functional class** | | 0.015 |
| 2 | 1 | |
| 3 | 3.41 (1.39-8.35) | 0.007 |
| 4 | 5.53 (1.32-23.10) | 0.019 |
| **Pack years** | 1.014 (1.002 – 1.026) | 0.020 |
| | | |
| **Laboratory parameters**: | | |
| C-reactive protein | 1.03 (1.02-1.04) | <0.001 |
| red cell distribution width | 1.12 (1.00 – 1.26) | 0.049 |
| | | |
| **Echocardiography variables**: | | |
| tricuspid annular plane systolic excursion | 0.86 (0.76-0.97) | 0.017 |
| | | |
| **6 Minute Walk Distance** | 0.993 (0.990 - 0.996) | **<**0.001 |
| | | |
| **Right heart catheterization** | | |
| systemic vascular resistance index | 1.000 (0.999 – 1.000) | 0.050 |
| ratio of pulmonary vascular resistance and systemic vascular resistance | 6.35 (1.30-31.1) | 0.023 |
| | | |
| **Cardiopulmonary Exercise Testing variables**: | | |
| peak oxygen uptake | 0.95 (0.91 – 1.00) | 0.048 |
| arterialized capillary partial pressure of oxygen at peak exercise | 0.95 (0.90 – 1.00) | 0.036 |

*Online supplements are not copyedited prior to posting and the author(s) take full responsibility for the accuracy of all data.*

**e-Table 6. Patient characteristics for subjects receiving vs. not receiving PAH specific treatment during any time of follow-up**

(PAH: pulmonary arterial hypertension, BMI: body mass index, FEV1: forced expiratory volume in the first second of expiration, FVC: forced vital capacity, TLC: total lung capacity, DLCOcSB: single breath diffusion capacity of lung for carbon monoxide corrected for hemoglobin, DLCOcVA: diffusion capacity of lung for carbon monoxide for alveolar volume corrected for hemoglobin, NT-proBNP: N-terminal pro brain natriuretic peptide, SPAP: systolic pulmonary arterial pressure, TAPSE: tricuspid annular plane systolic excursion, mPAP: mean pulmonary arterial pressure, PAWP: pulmonary arterial wedge pressure, PVR: pulmonary vascular resistance, PH: pulmonary hypertension, 6MWD: 6 minute walk distance, WU: Wood units)

Data are presented as median (interquartile range) or mean±SD, unless otherwise stated

| | no specific PAH treatment (n=80) | specific PAH treatment (n=62) | p value |
|---|---|---|---|
| **Age, years** | 67.4 (61.4 - 73.5) | 68.7 (63.6 – 73.1) | 0.56 |
| **Height, cm** | 170 (164 – 175) | 170 (165 – 176) | 0.23 |
| **Weight, kg** | 71 (61 – 87) | 78 (65 – 87) | 0.14 |
| **BMI, kg/m$^2$** | 24.8 (20.8 – 29.8) | 26.6 (22.6 – 29.8) | 0.23 |
| **Sex, N (%)** | | | |
| Male | 40 (50.0%) | 39 (62.9%) | 0.13 |
| Female | 40 (50.0%) | 23 (37.1%) | |
| **Systolic blood pressure** | 128 (119 – 144) | 119 (109 – 137) | 0.002 |
| **Diastolic blood pressure** | 69 ± 13 | 64 ± 12 | 0.028 |
| **Heart rate** | 73 (64 – 84) | 73 (66 – 84) | 0.85 |
| **WHO Functional class* (I/II/III/IV), N (%)** | 0 (0.0%)<br>33 (42.3%)<br>41 (52.6%)<br>4 (5.1%) | 0 (0.0%)<br>14 (22.5%)<br>45 (72.6%)<br>3 (4.8%) | 0.039 |
| **Pulmonary function test** | | | |
| $FEV_1$ (% predicted) | 49 (37 – 62) | 62 (48 – 74) | 0.002 |
| FVC (% predicted) | 72.5 ± 23.4 | 78.2 ± 18.0 | 0.11 |
| $FEV_1$/FVC (%) | 59 (51 – 64) | 62 (55 – 67) | 0.015 |
| TLC (% predicted) | 105 (93 – 123) | 103 (90 – 115) | 0.49 |
| DLCOcSB (% predicted) | 56.4 ± 20.5 | 53.5 ± 20.2 | 0.45 |
| DLCOcVA (% predicted) | 70.9 ± 25.9 | 65.3 ± 25.0 | 0.24 |
| GOLD stage of obstruction (I/II/III/IV), N (%) | 9 (11.3%)<br>31 (38.8%)<br>30 (37.5%)<br>10 (12.5%) | 9 (14.5%)<br>37 (59.7%)<br>10 (16.1%)<br>6 (9.7%) | 0.024 |

*Online supplements are not copyedited prior to posting and the author(s) take full responsibility for the accuracy of all data.*

| | | | |
|---|---|---|---|
| **Smoking habits, N (%)*** | | | |
| Current smokers | 10 (12.5%) | 7 (11.3%) | 0.78 |
| Pack-years | 15 (0 – 30) | 10 (0 – 30) | 0.74 |
| **Laboratory parameters** | | | |
| NT-proBNP (pg/ml) | 567 (161 – 1728) | 1712 (436 – 2804) | 0.002 |
| C-reactive protein (mg/l) | 5.0 (1.9 – 11.8) | 3.8 (2.2 – 11.1) | 0.88 |
| **Echocardiography** | | | |
| estimated SPAP (mmHg) | 58 (47 – 70) | 67 (56 – 81) | 0.004 |
| TAPSE (mm) | 21 (16 - 24) | 17 (14 - 21) | 0.033 |
| **Chest CT** | | | |
| mean pulmonary artery / ascending aorta diameter | 0.93 ± 0.16 | 1.02 ± 0.20 | 0.006 |
| mean pulmonary artery diameter (mm) | 32.4 ± 6.1 | 35.7 ± 5.6 | 0.005 |
| **Pulmonary hemodynamics** | | | |
| mPAP (mmHg) | 28 (24 – 38) | 41 (33 – 49) | <0.001 |
| PAWP (mmHg) | 10 (8 – 14) | 10 (7 – 12) | 0.47 |
| PVR (WU) | 3.63 (2.76 – 4.49) | 6.97 (4.60 – 9.61) | <0.001 |
| cardiac index (l/min/$m^2$) | 2.69 (2.33 – 3.19) | 2.35 (2.04 – 2.76) | 0.002 |
| systemic vascular resistance index (dyn s $cm^{-5}/m^2$) | 2307 (1914-2746) | 2433 (2036-2984) | 0.46 |
| **Exercise capacity** | | | |
| 6MWD (m) | 326 ± 113 | 271 ± 120 | 0.011 |
| peak oxygen uptake (% predicted) | 58 ± 21 | 45 ± 22 | 0.042 |

*information on WHO functional class and smoking status was available from n=140 subjects



*Online supplements are not copyedited prior to posting and the author(s) take full responsibility for the accuracy of all data.*
